# Outsourcing Agricultural Production: Evidence from Rice Farmers in Zhejiang Province

**DOI:** 10.1371/journal.pone.0170861

**Published:** 2017-01-27

**Authors:** Chen Ji, Hongdong Guo, Songqing Jin, Jin Yang

**Affiliations:** 1 China Academy of Rural Development, Zhejiang University, Hangzhou, Zhejiang, China; 2 Department of Agricultural, Food, and Resource Economics, Michigan State University, East Lansing, Michigan, United States of America; 3 Department of Economics, School of Economics, Huazhong University of Technology and Science, Wuhan, Hubei, China; Murdoch University, AUSTRALIA

## Abstract

China has recorded positive growth rates of grain production for the past eleven consecutive years. This is a remarkable accomplishment given that China’s rapid industrialization and urbanization has led to a vast reduction of arable land and agricultural labor to non-agricultural sectors. While there are many factors contributing to this happy outcome, one potential contributing factor that has received increasing attention is the emergence of agricultural production outsourcing, a new rural institution that has emerged in recent years. This study aims to contribute to the limited but growing literature on agricultural production outsourcing in China. Specifically, this study analyzes factors affecting farmers’ decisions to outsource any or some production tasks using data from rice farmers in Zhejiang province. Results from a logistic model show that farm size and government subsidy encourages farmers to outsource while ownership of agricultural machines and land fragmentation have negative effects on farmers’ decisions to outsource production tasks. Results also showed that determinants of outsourcing decisions vary with the production tasks that farmers outsourced.

## Introduction

China has food-provision responsibilities for one-fifth of the world’s population with less than one-tenth of the world’s arable land. Thus, food security has long been at the heart of development policies in China. China’s food security has also been increasingly challenged by rapidly changing economic and environmental landscapes. Specifically, rapid urbanization and industrialization processes, combined with environmental degradation, have caused arable land to shrink at alarming rates [1]. Furthermore, China continues to concurrently experience a steady flow of labor out of rural and into urban environments (i.e., from agricultural to non-agricultural sectors); according to data from the National Bureau of Statistics [2], China’s total number of migrants reached 261 million in 2010 [2].

While the loss of significant portions of arable land and agricultural labor might be expected to cause China’s grain production to sharply fall, China actually enjoyed positive grain-production growth each year from 2003 to 2014 [3]. This impressive growth (i.e., of China’s grain production over 11 consecutive years) has attracted the attention of scholars seeking to study factors that promote these positive outcomes, which include (i) agricultural policy and trade reform, (ii) human capital growth, (iii) price and market reform, (iv) public investment (e.g., in infrastructure and agricultural research), (v) institutional innovations (e.g., improvement of land tenure and the land rental market), and (vi) the emergence of agricultural cooperatives [4–10]. Also, an understudied, emerging institutional innovation that has, perhaps, played an important role in helping rural farmers overcome various agricultural production constraints is the agricultural-production outsourcing service (AOS). AOS is a type of contract arrangement; specifically, a farm household pays service fees to an individual or organization to complete one or more production tasks (plowing fields, sowing seeds, transplanting seedlings, and harvesting). It is worth noting that AOS studied in this paper is very different from the concept of “agricultural outsourcing’ (AO) that has recently been hotly studied in the international development literature [11–13]. AO is an outsourcing arrangement where capita-rich and natural resources-poor countries (e.g., China, South Korea, Japan, etc.) buy or lease huge quantities of arable lands from capita-poor and resources-rich countries (e.g., Sub-Saharan African countries) for the production of food or energy to support food or energy consumption of the guest countries [11–12]. Unlike AO which is a hot subject in international development, AOS is still a new development concept. In China, AOS is more popular in coastal provinces and regions where the challenges faced by agricultural sectors are more pronounced.

The literature on AOS, while scarce, has been growing in China in recent years. It is found that with the exception of a couple of related papers [16, 19], the majority of the papers on this topic are published in Chinese journals. The literature suggests several possible ways that AOS can affect crop production. 1) Agricultural-production outsourcing services support certain labor-constrained households (e.g., with migrant or local off-farm workers) to continue to engage in crop production and farming on their own land [14–15]. 2) Agricultural-production outsourcing services also support farmers who lack sufficient agricultural machinery via the outsourcing of agricultural machinery services [15–16]. Since machine ownership is found to be positively correlated with farm size [17], agricultural-production outsourcing is more likely to help small-scale farms overcome small-farm disadvantages associated with machine use. 3) Agricultural-production outsourcing services can also assist farmers who lack certain key skills to overcome such constraints [18]. 4) The outsourcing of agricultural production can also be expected to lower costs and increase profits via various means (e.g., work specialization and economies of scale) [18].

This study contributes to the emerging literature on agricultural-production outsourcing and identifies factors that affect the decisions of farmers to outsource agricultural production using data from rice farmers in Zhejiang Province. Results from a logistic model indicate that farm size and government subsidies tend to encourage farmers to outsource rice production while labor endowment, ownership of agricultural machinery, and (to some extent) land fragmentation are negatively associated with the decisions of farmers to outsource. Results also suggest that agricultural-production outsourcing decisions (by farmers) are correlated with specific production tasks outsourced by farmers. Thus, this paper makes two significant contributions to the literature. First, it is among the first few studies to bring this important institution to the attention of international communities. Second, it utilizes an ideal setting: Zhejiang Province, which is one of the most urbanized and industrialized provinces with traditional importance in rice production. We will provide a more detailed description of this province in the next section.

The rest of this paper is organized as follows: Section 2 briefly discusses the history of rice production in Zhejiang Province. Section 3 develops a number of hypotheses based on a brief theoretical discussion. Data, summary statistics, and empirical methods are discussed in Section 4. Econometrics results are discussed in Section 5. Section 6 concludes with some policy implications.

## Rice Production in Zhejiang

Zhejiang Province is located on the East Coast of China and was one of the major grain-production bases in the pre-reform era. Since the beginning of China’s rural reforms in 1978, Zhejiang Province has consistently been one of the front-runners in economic development; however, the unprecedented pace and scale of industrialization and urbanization in Zhejiang Province have resulted in substantial reductions of arable land. According to recent statistics, total crop area and production have been reduced by 63.9% and 46.6% (from 1978 and 2012). By the end of 2012, the average crop production area per capita in Zhejiang Province was 0.023 hectare (ha), the crop self-sufficiency ratio was less than 40%, and the gap between crop demand and supply was 1.2 million tons; thus, this province now has the second-largest food deficiency in the nation [3]. Therefore, local and provincial governments are now frequently interested in policies that can potentially help stabilize and promote crop production.

In Zhejiang Province, rice is the main staple food of Zhejiang citizens and has been grown for centuries. In terms of production scale, rice-production entities in Zhejiang Province can be divided into five categories: small-scale farms (<20 mu or 1.3 ha), large-scale farms (20–100 mu), large commercial farms (>100 mu), rice cooperatives, and specialized rice-production companies. The composition of these five categories is evolving over time: (i) both the sizes and numbers of small-scale farms have dropped considerably over time, (ii) large-scale farms have experienced reductions in production scales (but not in numbers), and (iii) the numbers and production scales of large commercial farms and grain cooperatives have increased. (However, there are still very few grain companies with enormous production scales.) By 2012, percentages associated with each of the five categories, from small to large (in total area), is 66%, 7.2%, 16.5%, 10.2%, and 0.6%, respectively. Thus, despite the trend of shifting from small- to large-scale farms, small-scale farms with farm sizes less than 1.3 ha remained the dominant rice-production entity in Zhejiang (as of 2012).

A brief discussion follows on the general characteristics of the first three types of rice producers. We concentrate on these three types of farms because our analyses are based on data from household-based farms only; together, they represent approximately 90% of the total rice sown area in Zhejiang Province. Rice cooperatives and companies (i.e., the last two categories) are not household-based farms; thus, their decisions to outsource agricultural production are likely to significantly differ from household-based rice producers.

Operators of small-scale farms are typically in their 60s, produce rice on their own farmland (or on the farmland of relatives or friends) with few or no rental fees, traditionally produce rice for their own consumption (vs. for the generation of income), are experienced rice farmers, and tend to rely on others to provide harvesting and plowing services. Operators of large-scale farms are typically in their 50s, rent land from small-scale farmers or village communities (to expand their rice-production scales), are skillful rice farmers, generate most of their household income from rice production, receive government subsidies for growing rice on large scales, typically own small agricultural machinery, often provide some agricultural services (e.g., plowing) to small-scale farms, and need to expand (or at least retain) their scale to attain economies of scale; otherwise, they will shift from producing rice to working in cities. Operators of big commercial farms have generally retired, previously operated their own businesses (or worked as employees in urban areas), purchased or rented land from village communities or individual farmers, and hire workers to work on their farmland. They are motivated by local policies that encourage development of commercialized rice farms and mainly sell rice to governments via contracts.

Zhejiang Province is an ideal setting to study agricultural-production outsourcing services for a myriad of reasons. 1) These services have been widely adopted by rice farmers who outsource many production tasks (e.g. land plowing, sowing of seeds, germinating of seedlings, seedling transplantation, crop protection, harvesting, and post-harvesting activities). 2) Zhejiang Province is one of the most developed coastal provinces with vibrant rural and non-farm economies and thus has a significant demand for (and supply of) agricultural-production outsourcing services. The high demand for outsourcing service is due to the fact that farmers face abundant employment opportunities in non-farm sectors; thus the opportunity costs of working on farms are quite high. Meanwhile, there is a significant supply of outsourcing services due to the considerable support of provincial and local governments for scale farming and agricultural cooperatives. 3) Rice has a long crop season with labor-intensive tasks during some key phases (e.g., land plowing, seedling transplantation, and harvesting) and technique-intensive tasks in other phases (e.g., seedling nursery and plant protection); for this reason, most of the existing studies by Chinese scholars almost exclusively focus on rice [18]. 4)The coexistence of different types of farms with large variation in farm sizes allows us to compare determinants of agricultural-production outsourcing across farm scale, which can help guide the future development of agricultural outsourcing (due to vibrant changes in farm structure over time).

## Theoretical Discussion and Hypotheses

As outsourcing agricultural production is not a common phenomenon in other countries, there is a paucity of literature on agricultural-production outsourcing outside of China. However, there are increasing numbers of studies by Chinese researchers on determinants associated with decisions to outsource agricultural production in China. Existing studies in the literature support the notion that farmers are rational economic agents; thus, their decisions to outsource particular production tasks are affected by factors related to the benefits and costs of the two competing options (i.e., outsourcing or not-outsourcing). Farmers incur service fees when they choose to outsource; however, they, can also save time, reduce operational costs, and spend time on earning income from other activities. If the benefits outweigh the costs of outsourcing, they will choose to outsource; however, if the costs outweigh the benefits, they will choose not to outsource.

Thus, farmers are likely to contemplate factors affecting the benefits and costs of outsourcing agricultural production when making outsourcing decisions. Against this background, we are proposing the following hypotheses that will be tested empirically using household survey data:

Hypothesis 1: Rice farmers with larger production scales are more likely to outsource rice production than those with smaller production scales.

The reasons why farmers with larger production scales are more likely to outsource agricultural production than those with smaller production scales are quite intuitive. First, one of the main challenges facing China’s food security is the rapid rise of wage rate and the increasing shortage of agricultural labor, as suggested by the finding that China has reached the Lewis Turning Point [20]. In the extreme case, farmers may not be able to hire sufficient agricultural labor at any cost at a certain production season, which could cause serious loss of production. And more generally, the cost of labor may be higher than the cost of outsourcing the same task to a service provider. It is plausible that the labor shortage problem tends to be much more acute for farmers with larger production scales than those with smaller production scales, especially during the peak crop seasons (e.g., land preparation, transplanting and harvesting seasons). Therefore, holding constant all other determinants (i.e. outsourcing price, government subsidy, machinery ownership, labor endowment, and technology), larger farmers are more likely to outsource rice production than small farmers. Studies have found that in production tasks such as sowing of seeds, transplanting and pests’ prevention, farmers of larger production scales are much more likely to outsource than those of smaller scale [15]. Theoretical and empirical evidence from India has also shown that in response to the rapid wage rise, large scale farmers use much less labor per acre, and are more mechanized than smaller scale farmers [21]. Second, from the supply side, the outsourcing service providers are less willing to offer their services to smaller farmers out of the concern that a certain level of scale is necessary to achieve the economies of scale of the outsourcing service operation.

Hypothesis 2: Rice farms with significantly fragmented farmland are less likely to outsource rice production.

The logic underlying this hypothesis goes: For a given land area, the operational cost of agricultural equipment (e.g., tractor, harvester, etc.) is smaller when the land is in one piece than when it is fragmented into multiple pieces. It is argued that fragmented land prevents farmers from outsourcing rice production because machinery asset does not work well on fragmented lands, and using machines on fragmented lands does not allow any service provider to achieve economies of scale [22]. This same point is discussed by others [8, 21].

Hypothesis 3: Rice farmers with more working members are less likely to outsource agricultural production.

Again, this hypothesis is intuitive. Rice farmers with more working members are less likely to be labor constrained. Therefore, holding everything else constant, rice farmers with more family labor are more likely to complete certain production tasks by themselves than those with less family labor, especially for the labor-intensive production tasks.

Hypothesis 4: Government subsidies have positive effects on the decisions of rice farmers to outsource rice production.

This hypothesis is motivated by the basic price theory. As the price of outsourcing service is higher, the demand for outsourcing services would be smaller. The government subsidy for outsourcing services is equivalent to reduce (increase) the price of outsourcing service that rice farmers pay (and supply providers receive). It is obvious that the more subsidies that a government provides to farmers, the more likely they will choose to outsource agricultural production.

Hypothesis 5: Rice farms who possess more agricultural machinery are less likely to outsource rice production.

The reason why rice farmers with more agricultural machinery are less likely to outsource is straightforward. When farmers own agricultural machinery, they tend to use their own agricultural machinery to accomplish rice-production tasks; thus, holding other things constant, farmers who have their own agricultural machinery are less likely to outsource.

Hypothesis 6: Farmers’ migrant experiences have positive influence on rice farmers to outsource rice production.

Finally, farmers who have comparative advantages in local off-farm employment and/or migration are more likely to outsource rice production tasks (especially for those labor intensive ones). Migrant members are either unable to come back to work on their farm (e.g., employers not permitting) or the cost of doing so is too high (e.g., loss of current jobs). Hence, it is to their economic interest to outsource rice production to allow them to stay in the destination locations without causing any disruption in job security.

In the following section, we will discuss the sample, data and the method used to test the hypotheses empirically using our survey data.

## Survey Design, Data Description, and Empirical Method

**Fig 1:**
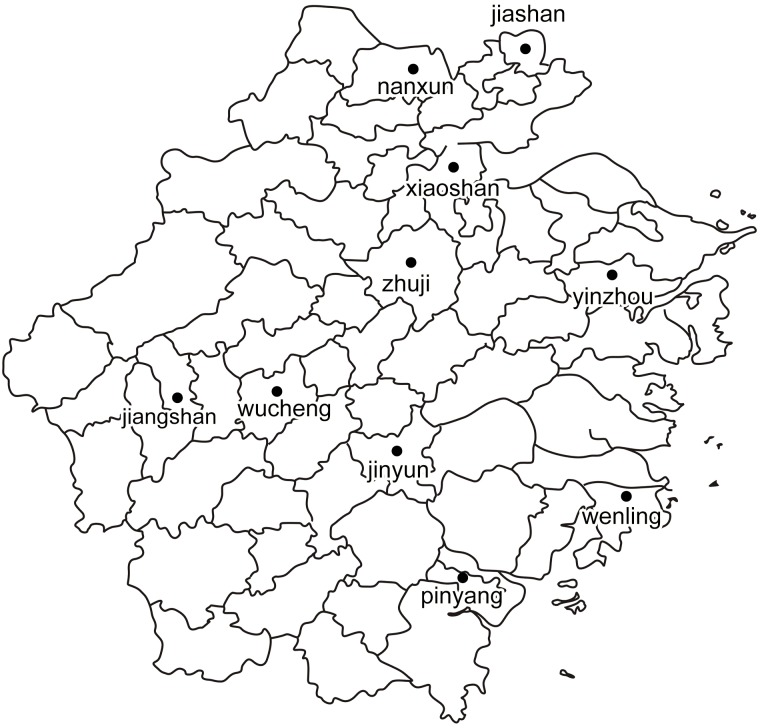
Geographical distribution of the 10 sample counties.

A household survey was jointly conducted by the Zhejiang Department of Agriculture’s Crop Bureau, Zhejiang University, and Zhejiang Normal University from December 2012 to January 2013. Ten counties (five in southern Zhejiang and five in northern Zhejiang) from 10 different prefectures were selected as our sample counties. And they are: Xiaoshan (Hangzhou prefecture), Jiashan (Jiaxing prefecture), Nanxun (Huzhou prefecture), Yinzhou (Ningbo prefecture), and Zhuji (Shaoxing prefecture); the five counties in southern Zhejiang include Wenling (Taizhou prefecture), Pingyang (Wenzhou prefecture), Wucheng (Jinhua prefecture), Jiangshan (Quzhou prefecture), and Jinyun (Lishui prefecture). Fig 1 shows the geographical distribution of the 10 sample counties in Zhejiang Province. In each county surveyed, we selected 20 small-scale rice producers and 10 large-scale rice producers randomly. Thus we collected 300 rice producers of the whole sample, and finally 271 survey questionnaires were effective as 29 were dropped due to incomplete information.

Before the real field interview started, questionnaires were carefully and purposively designed through multiple rounds of discussions and field tests; also, student enumerators were given instructions on how to conduct interviews. The study including the survey instrument and the survey sample was reviewed and approved by the Research Committee of Zhejiang Modern Agriculture under Zhejiang Department of Agriculture. The information collected from the survey was not sensitive and kept unidentifiable. Verbal consent was obtained from each participant prior to the interview. The justifications for using verbal consent include the following: (1) Oral consent is common for household surveys in China that do not involve sensitive information; (2) during the pre-test, we found out that a significant number of respondents were illiterate and could not even sign their names; (3) the Research Committee of Zhejiang Modern Agriculture under Zhejiang Department of Agriculture considered our survey of low risk to survey households and approved our proposal to use oral consent.

Table 1 shows the distribution of the 271 surveyed rice producers in the 10 sample counties. Before we econometrically analyze the determinants of rice farmers’ decisions to outsource rice production tasks, we first conduct a descriptive analysis to generate a better understanding of key demographic, economic, and agricultural production characteristics as well as outsourcing behaviors associated with the different types of rice farmers in our sample.

**Table 1:** Number of sample households in each sample county.

Prefecture	County	Sample amount	Percentage (%)
Jiaxing	Jiashan	25	9.22
Huzhou	Nanxun	24	8.86
Hangzhou	Xiaoshan	16	5.90
Ningbo	Yinzhou	20	7.38
Shaoxing	Zhuji	27	9.96
Quzhou	Jiangshan	42	15.50
Li’shui	Jinyun	31	11.44
Wenzhou	Pingyang	30	11.07
Taizhou	Wenling	28	10.33
Jinhua	Wucheng	28	10.33
Total		271	100

Source: Own computation based on own household survey conducted in 2012.

### Demographic Characteristics and Asset Ownership of Sample Rice Farms

Table 2 presents the demographic and economic characteristics of rice farms in our sample. Our data show that the average size of a rice farm household is 4.4 with 3.4 working members. The age of an average household head (who is in charge of rice production) in our sample is 53. More than 85%of heads of households have received primary school education; half of the heads are graduates of secondary schools. Roughly 30% of heads are village leaders and 30% are party members.

**Table 2:** Mean household characteristics in the sample.

Items	Mean	Min	Max	N	S.d
**General characteristics:**					
Household size	4.40	1	12	268	1.70
Number of working members	3.40	1	8	268	1.30
Head’s age	52.8	24	82	267	9.40
Head not finishing primary school	0.14	0	1	269	0.35
Head completed primary school	0.35	0	1	269	0.48
Head completed middle school	0.35	0	1	269	0.48
Head completed high school (or higher)	0.11	0	1	269	0.31
Head is a village leader	0.34	0	1	268	0.50
Head is a party member	0.33	0	1	267	0.50
**Employment and asset ownership:**					
Cultivated land area (mu)	120 (28.50)*	0	1100	260	196.20
Number of plots	4.00	0	100	268	7.50
Number of tractors owned	0.82	0	8	251	1.29
Number of plowing machines owned	0.32	0	4	248	0.80
Number of transplantation machines owned	0.66	0	8	251	1.46
Number of combined harvesters owned	0.39	0	6	250	0.90
Subsidy received per mu of land (RMB)	132.00	0	525	183	0.00
Past migration experience	0.26	0	1	268	0.44
Number of observations	269	-	-	-	-

Note:

* The number inside the parenthesis is the median value of the cultivated area (mu)

Source: Own computation based on own household survey conducted in 2012

There is a big variation in cultivated land area and the number of plots across farms. The mean value and the median value of the cultivated land area of our sample are 120 mu and 28.5 mu, respectively. If we divide the sample into three even groups according to cultivated land area, the mean cultivated land area for small, medium, and large farms is 3.3 mu, 56 mu, and 493 mu, respectively. To ensure there is a sufficient variation in farm scale to test for the effect of farm scale on farmers’ outsourcing decisions, we oversampled large rice farms. As a result, the average farm size of our sample appears much bigger than a typical rice farm in Zhejiang Province. To see how much our econometrics results are driven by the subsample of these large rice farms. In a separate regression (not reported), we tried the same regression without the large farm group, the results are largely consistent with those based on the full sample. The median cultivated land area of the sample farms is 28.5 mu, which is a better representation of a typical rice farm’s production scale in Zhejiang province. The sample farms have an average 4 plots with the standard deviation of 7.5 plots. In terms of farm assets, the most popular assets are tractors, plows, transplantation machines, and combined harvesters. An average farm owns 0.8 tractors (from 0 to 8), 0.32 (from 0 to 4) plows, 0.66 (from 0 to 8) transplantation machines, and 0.39 (from 0 to 6) combined harvesters. The utilization of subsidies is common; 94.4% of households received subsidies during the study period. The average value of the subsidy received is 132 RMB (≈US$ 21) per mu of land.

### Demand and Supply of Rice Production Outsourcing Services

Table 3 presents the use of outsourcing services for each individual production task by (i) farm size (Columns 2–4) and (ii) labor endowment (Columns 5–7). A few interesting observations emerge from this table. First, harvesting is the most commonly outsourced task: 74% of households in the small-size land group and 80% of households in both the medium- and large-size land groups outsourced this task. Plowing is the second-most outsourced task followed by the plant-protection task. In the case of plowing and harvesting, we further note that outsourcing is more common among small (vs. large) farms; this could partly be reflected by the fact that large farms own more agricultural machinery. For other tasks (e.g., sowing of seeds, seedling transplanting, and plant protection), it is evident that the likelihood of outsourcing is greater for land groups deemed medium and large (vs. small) in size. For a number of tasks, however, the difference in the likelihood of outsourcing between land groups considered large and medium (in size) was not always consistent with expectations. The picture is also mixed when it comes to the relationship between the use of outsourcing services and household labor endowments. However, the mixed evidence does not necessarily contradict with our hypotheses because the descriptive analysis presented in Table 3 does not control for any other factors affecting the outsourcing decisions of farmers. We will need to conduct a multivariate analysis to perform hypothesis testing.

**Table 3:** Use of agricultural outsourcing services by land size and labor endowment (%).

	Whole sample	By land			By labor endowment		
		Small	Medium	Large	Small	Medium	Large
Plough	220	0.68	0.55	0.58	0.61	0.57	0.66
Seeding	220	0.32	0.39	0.38	0.36	0.42	0.26
Transplanting	220	0.30	0.50	0.43	0.43	0.46	0.26
Plant protection	220	0.55	0.58	0.60	0.55	0.57	0.71
Harvest	220	0.80	0.80	0.74	0.73	0.84	0.80
No. of Observations	269	74	74	72	110	74	35

Source: Own computation based on own household survey in 2012.

Table 4 presents the outsourcing services provided by different agents for each production task. Overall, rice-production cooperatives (36.2% for plowing to 72.1% for plant protection) and large rice producers (9.1% for plant protection to 46.2% for harvesting) are by far the most important providers (accounting for 75%, or higher, share of all the sourcing services provided for almost all of the individual tasks). The rest of the outsourcing services are provided by relatives and friends (ranging from 5.8% for seeding to 9.2% for plowing), government agricultural departments (ranging from 2% for harvesting to 11.7% for plant protection), and agricultural service companies (ranging from 0.65% for plant protection to 4.9% for plowing).

**Table 4:** Agricultural Outsourcing Services by Various Service Providers (%).

	Big rice producer	Rice production cooperative	Agricultural service companies	Relative & friends/ community	Government agricultural department
Plowing	44.79	36.20	4.91	9.20	4.91
Sowing of Seeds	11.54	70.19	2.88	5.77	9.62
Transplanting Seedlings	19.33	65.55	2.52	5.88	6.72
Plant protection	9.09	72.08	0.65	6.50	11.69
Harvesting	46.23	43.72	2.01	6.03	2.01

Source: Own computation based on own household survey in 2012.

### Empirical Method

While such descriptive analysis based on a simple tabulation is informative, it requires multivariate regressions to identify the multiple factors that can jointly determine the outsourcing decisions of farmers. As discussed in the previous section, all factors that potentially affect the benefits and costs of outsourcing (or not outsourcing) should be included in the regression analysis. Discrete choice model (i.e., probit or logit) is a standard model utilized to analyze determinants associated with the decisions of farmers to outsource agricultural-production tasks. We adopt the logit model in our analysis, and we also estimate a probit model for robustness check (not reported). As expected, the results are highly consistent with those from the logistic model.

The logit model estimates the probability that a farmer might outsource, as a function of all the factors that could potentially affect the farmer’s decision to outsource; specifically, we will have

$$P\left(y_{ji}=1|x_{1i},x_{2i},\;\ldots ,x_{ki}\right)=\frac{\text{exp}\left(\beta_{0}+\beta_{1}x_{1i}+\beta_{2}x_{2i}+\ldots +\beta_{k}x_{ki}\right)}{1+\text{exp}\left(\beta_{0}+\beta_{1}x_{1i}+\beta_{2}x_{2i}+\ldots +\beta_{k}x_{ki}\right)}$$ (1)

In Eq (1), *y*_*ji*_ is a dependent binary variable (= 1 if farmer *i* outsource task *j*, = 0 otherwise), *P*(.) is the probability for farmer *i* to outsource task *j* (or the probability for *y*_*ji*_ = = 1), *x*_1*i*_, *x*_2*i*_, …, *x*_*ki*_ are the independent variables that are expected to influence *P*(.).*β*_0_, *β*_1_,…, *β*_*k*_ are the coefficients of the independent variables to be estimated. Specifically, the independent variables include farm-scale dummies (Hypothesis 1), number of plots as a measure of fragmentation (Hypothesis 2), family labor-endowment dummies (Hypothesis 3), subsidies received (Hypothesis 4), ownership of different agricultural machineries (Hypothesis 5), migration experience, and other household-level control variables (e.g., ages and education levels of heads of households). A more detailed explanation of the independent variables is provided in Table 2.

Eq (1) can be easily estimated by Maximum Likelihood Estimation (MLE) for each individual production task. Although not reported, the probit model is also estimated; the results from the probit model are consistent with those from the logit model. Since households can outsource more than one production task, we also utilize a Poisson regression to estimate the determinants of outsourcing intensity (i.e., total number of production tasks outsourced).

The survey also collected information on the willingness of farmers to outsource rice production tasks in the future; thus, Eq (1) is also used to estimate determinants of the willingness of farmers to outsource agricultural-production tasks in the future. To implement this analysis, we replace the dependent variable in Eq (1) by *household’s willingness to outsource* each of the five agricultural-production tasks (i.e., *y*_*ji*_ = 1 if household *i* is willing to outsource task *j* in the future, = 0 otherwise). The right-hand side variables are exactly the same as before.

## Results and Discussions

Table 5 reports econometrics results on determinants of using outsourcing services (Columns 1–5) and adoption intensity (Column 6) during the survey year. The logit model is estimated for each of the five main production tasks (i.e., plowing, sowing of seeds, transplanting, plant protection, and harvesting) with each column corresponding to one of the production tasks. In all of the regressions, county dummy variables are included; the standard errors of the estimated coefficients are all adjusted for possible clustering effects at the village level. To ease the interpretation of the results, the reported coefficients for each variable (*x*_*ki*_) in the logit models are all marginal effects (∂*P*(*y*_*ji*_ = 1)/∂*x*_*ki*_). The econometrics analysis yields a number of interesting results and many are consistent with our hypotheses. The relative significances of different determinants, however, vary by particular production tasks that are outsourced.

**Table 5:** Logit model results on farmers’ decisions to outsource agricultural production tasks.

	Plowing	Sowing of Seeds	Rice Transplanting	Plant Protection	Harvesting	No of tasks outsourced
	(1)	(2)	(3)	(4)	(5)	(6)
Small-scale farm	-0.087	-0.361**	-0.547***	-0.205	-0.197*	-0.827***
	(0.11)	(0.18)	(0.19)	(0.17)	(0.11)	(0.31)
Big-scale farm	0.260**	0.092	0.242**	0.341***	0.041	0.395
	(0.13)	(0.12)	(0.12)	(0.13)	(0.07)	(0.42)
Small labor endowment	0.399	0.786***	0.645**	0.628**	0.107	1.777***
	(0.26)	(0.21)	(0.27)	(0.25)	(0.10)	(0.34)
Large labor endowment	0.067	-0.183	-0.273***	0.137	-0.025	0.271
	(0.09)	(0.12)	(0.09)	(0.11)	(0.07)	(0.42)
Number of plots	-0.113	0.048	-0.071	-0.142*	-0.032	-0.11
	(0.11)	(0.11)	(0.10)	(0.08)	(0.08)	(0.21)
Government Subsidy	0.670**	0.438*	0.146	0.329	0.416***	0.991
	(0.28)	(0.24)	(0.33)	(0.29)	(0.15)	(0.72)
No. of tractors	-0.061	0.042	0.007	0.094	0	-0.048
	(0.04)	(0.07)	(0.05)	(0.07)	(0.03)	(0.19)
No. of plowing machines	-0.111	0.072	0.142**	-0.034	-0.023	-0.018
	(0.07)	(0.08)	(0.07)	(0.05)	(0.03)	(0.16)
No. transplanting machines	0.011	-0.065***	-0.134***	-0.108***	0.024	-0.072
	(0.04)	(0.03)	(0.04)	(0.04)	(0.02)	(0.08)
No. of combine harvesters	-0.088	-0.034	-0.067	-0.032	-0.212***	-0.096
	(0.07)	(0.05)	(0.04)	(0.08)	(0.06)	(0.25)
Head’s age	0.001	-0.009	-0.017**	-0.015**	-0.001	-0.033*
	(0.01)	(0.01)	(0.01)	(0.01)	(0.00)	(0.02)
Head’s education	-0.006	0.036*	0.01	0.01	0	0
	(0.02)	(0.02)	(0.02)	(0.02)	(0.01)	(0.06)
Village leader dummy	-0.034	0.088	0.138	-0.062	-0.034	0.367
	(0.13)	(0.12)	(0.13)	(0.11)	(0.08)	(0.31)
Party member dummy	-0.101	-0.14	-0.06	0.073	-0.124**	0.136
	(0.09)	(0.13)	(0.12)	(0.14)	(0.06)	(0.31)
Migration experience	0.027	0.017	-0.106	-0.013	0.072	-0.138
	(0.10)	(0.08)	(0.07)	(0.12)	(0.07)	(0.35)
County fixed-effect	yes	Yes	yes	yes	yes	yes
Clustering effect at the village level corrected	yes	Yes	yes	yes	yes	yes
Number of observations	204	176	192	168	196	204
Value of log-likelihood	-102.034	-86.119	-84.513	-89.206	-75.564	-82.226

Notes

*, ** and *** are significant at 10%, 5% and1%, respectively.

The base farm scale group is the medium farm scale dummy

The base labor endowment group is the medium labor endowment

### Determinants for Outsourcing Services in the Survey Year

First, the results in the first two rows support Hypothesis 1, which is that (holding other things constant) large farms are more likely to outsource agricultural-production tasks. In a comparison with medium-scale farms (i.e., the base group in the regression), the coefficient on the large-scale (small-scale) farm dummy variable is positive (negative) throughout production tasks and statistically significant in three out of five production tasks. While we included as many variables that could affect farmers’ decisions to outsource agricultural production tasks as possible, it is still possible that some unobserved factors (e.g., farming ability) are potentially correlated with production scales and farmers’ decisions to outsource production tasks. If we can assume that farming ability is positively correlated with both production scale and farmers’ outsourcing decisions, then failing to control for farming ability would underestimate the coefficient of production scale. In other words, if the farming ability were controlled, farmers with large scale production would be even more likely to outsource agricultural production. Moreover, farm scale seems to be particularly important for the rice-transplanting task (relative to the five production tasks); here, both small- and large-scale farm dummy variables are significant at 5% (Column 3), whereas only one of the two dummy variables is significant for all of the other tasks. Relatedly, land fragmentation also matters. The coefficient associated with the number of scattered plots (a measure of fragmentation) has negative sign for all but one case (i.e., sowing of seeds). The negative sign is expected because it is more difficult and costly to perform outsourcing services (Hypothesis 2). However, the coefficient is statistically insignificant for four of the five tasks except for the task of plant protection where it is significant at 10%; this prevents us from drawing a strong conclusion. The insignificance could be due to a relatively small sample size and small variation in the number of plots.

Second, the results also tend to support the argument that agricultural-production outsourcing helps overcome labor constraints (Hypothesis 3), especially for those tasks that require more intensive labor input. The coefficient on small labor-endowment dummy (medium labor-endowment dummy omitted as the base group) is positive throughout all production tasks and statistically significant in cases of (i) sowing of seeds, (ii) rice transplanting, and (iii) plant protection (Row 3). This suggests that farms with labor constraints are more likely to outsource production tasks (especially for traditionally labor-intensive tasks). The coefficient of the large labor-endowment dummy is negative and statistically significant only in the case of rice transplanting; it is insignificant (with mixed signs) for all other tasks (Row 4), reinforcing the argument that farms with the smallest labor endowments are more likely to use outsourcing services.

Third, the government subsidy on rice production is positively correlated with the probability of farmers adopting outsourcing services across all five tasks (Row 6). Ideally, we would also like to have price information on the outsourced services. Due to the lack of reliable price information, we are not able to directly test the price effect on the use of outsourcing services. County-dummy variables are included in all of the regressions so the biases (caused by omitted price information) are reduced if we can assume price does not vary much within a county. The positive coefficient on subsidy dummy is also statistically significant in cases of plowing, sowing of seeds, and harvesting; this indicates that when farmers receive government subsidies for growing rice, they are more economically able to outsource (which is consistent with Hypothesis 4).

Fourth, the econometrics results also confirm the importance of the ownership status of agricultural machinery (i.e., in the outsourcing decisions of farmers). There is a negative correlation between farmers who own more planting machines and the use of outsourcing services with sowing of seeds and rice transplanting (significant at the 1% level) since they can use these machines to complete these tasks themselves (Hypothesis 5). Furthermore, farmers who own more harvesting machines are less likely to outsource harvesting services (significant at the 1% level), meaning that farmers who have more harvesting machines are able to perform the tasks by themselves. The numbers of tractors and plows have expected negative coefficients in relation to plowing; however, neither is significant (which can be explained by the fact that these machines may be used for other purposes).

Fifth, in terms of other household control variables, the decisions of rice farmers to outsource are unrelated to the education levels and political positions of heads of households; however, the negative and significant coefficient on age (of the head of household), associated with rice-planting and plant-protection tasks, suggests that younger rice farmers are more likely to outsource. The migration experiences of heads of households have no effect on their outsourcing decisions, which is somewhat unexpected.

Finally, for robustness check, we also employed a Poisson regression to estimate the determinants of the number of rice-production tasks outsourced. The results from the Poisson regression are generally consistent with those from the logit models (Column 6). First, the coefficients have the expected signs for all key variables (i.e., a positive sign for large-scale farms, small labor-endowment dummy, and government subsidies and negative signs for small-scale farms, large labor-endowment dummy, fragmentation, and all of the agricultural machinery variables). Second, the coefficients of the small-scale farm dummy and small labor-endowment dummy are also statistically significant at the 1% level, further reinforcing (i) the importance of the limiting effect of small-scale farms on participating in outsourcing services and (ii) the potential role of outsourcing services in helping certain rice farmers overcome labor constraints.

### Determinants of Willingness to Use Outsourcing Services in the Future

The results on determinants of willingness to use outsourcing services in the future are reported in Table 6. The results are mostly similar to those reported in Table 5, regardless of whether they are based on logit models (Columns 1–5) or on the Poisson model (Column 6). The results again suggest that farm scale, labor endowment, government subsidies, and ownership of agricultural machinery are the most important determinants of perceived future adoption of outsourcing services. The results of these variables largely support the hypotheses: (i) small scale farm households are less likely to outsource, (ii) farm households with smaller (vs. medium or large) labor endowments are more likely to outsource, (iii) households receiving subsidies are more likely to outsource, and (iv) farm households with more agricultural machinery are less likely to outsource.

**Table 6:** Logit model results on farmers’ willingness to adopt outsourcing services in the future.

	Plowing	Sowing of Seeds	Rice transplanting	Plant protection	Harvesting	No. of tasks outsourced
	(1)	(2)	(3)	(4)	(5)	(6)
Small-scale farm	-0.05	-0.364**	-0.576***	-0.221*	-0.145	-0.719**
	(0.13)	(0.18)	(0.18)	(0.13)	(0.12)	(0.34)
Big-scale farm	-0.052	0.006	0.037	-0.037	-0.055	-0.252
	(0.11)	(0.14)	(0.13)	(0.09)	(0.09)	(0.41)
Small labor endowment	0.374	0.575***	0.832***	0.419**	0.036	1.225***
	(0.25)	(0.21)	(0.25)	(0.21)	(0.14)	(0.21)
Large labor endowment	-0.055	-0.151	-0.300**	0.053	-0.032	0.083
	(0.10)	(0.12)	(0.15)	(0.14)	(0.09)	(0.30)
Number of plots	0.002	0.111	-0.078	-0.032	-0.036	0.121
	(0.10)	(0.11)	(0.11)	(0.09)	(0.09)	(0.19)
Government Subsidy	0.530**	0.139	0.492**	0.129	0.317*	0.285
	(0.21)	(0.17)	(0.24)	(0.20)	(0.16)	(0.42)
No. of tractors	-0.070*	-0.001	-0.025	0.042	-0.029	-0.223**
	(0.04)	(0.05)	(0.05)	(0.04)	(0.03)	(0.10)
No. of plowing machines	-0.076*	-0.03	-0.011	-0.081	-0.025	0.001
	(0.04)	(0.08)	(0.08)	(0.07)	(0.05)	(0.13)
No. transplanting machines	0.022	-0.049	-0.082**	-0.026	0.037	-0.005
	(0.04)	(0.04)	(0.04)	(0.04)	(0.04)	(0.07)
No. of combine harvesters	0.028	0.007	-0.03	-0.018	-0.111***	0.176
	(0.05)	(0.07)	(0.06)	(0.05)	(0.04)	(0.12)
Head’s age	0.004	-0.005	-0.015***	-0.006	0	-0.013
	(0.01)	(0.01)	(0.01)	(0.01)	(0.01)	(0.01)
Head’s education	0.009	0.061**	0.019	0.026	0.012	0.088*
	(0.02)	(0.03)	(0.02)	(0.03)	(0.02)	(0.05)
Village leader dummy	-0.015	-0.128	-0.128	-0.077	-0.071	0.036
	(0.08)	(0.12)	(0.16)	(0.11)	(0.10)	(0.21)
Party member dummy	-0.138**	-0.237**	-0.165	-0.096	-0.109	-0.342
	(0.05)	(0.11)	(0.12)	(0.11)	(0.10)	(0.24)
Migration experience	-0.071	-0.057	-0.065	-0.073	0.001	-0.105
	(0.09)	(0.07)	(0.06)	(0.08)	(0.07)	(0.22)
County fixed-effect	Yes	Yes	yes	yes	yes	yes
Clustering effects at village level corrected	Yes	Yes	yes	yes	yes	yes
Number of observations	204	192	192	204	204	204
Value of log-likelihood	-113.513	-95.035	-92.711	-114.415	-109.667	-114.371

Notes

*, **,and *** are significant at 10%, 5% and1%, respectively.

The base farm scale group is the medium farm scale dummy

The base labor endowment group is the medium labor endowment

Despite the highly consistent results between Tables 5 and 6, there is one noticeable exception that is worth some discussion. Unlike Table 5 where the coefficient on big-scale farm is positive across all the six regressions (columns 1–5 corresponding to five tasks and column 6 for the Poisson regression) and statistically significant in three cases, the coefficient on the big-scale farm in Table 6 is insignificant in all cases and even negative in four out of six cases. The change of the coefficient of big-scale farm from ‘significant’ in Table 5 (regressions of the current outsourcing decisions) to ‘insignificant’ in Table 6 (regressions of hypothetical outsourcing decisions in the future) may mean that being a big-farm was not rewarded in terms of scale advantage (of using the outsourcing services) as much as originally expected.

While the dependent variable in Table 6 is a perceived future decision of outsourcing, the associated results are unlikely to be subject to the same simultaneity bias as those reported in Table 5 because the future decision is unlikely to have effects on the right-hand side variables that are measured in the current time. The fact that the results are highly consistent in both tables further increases our confidence about the main results of the study.

## Conclusion

China’s rapid urbanization and industrialization will continue to create increasing pressure on the nation’s food security. The attainment of food security in this extremely challenging situation will be prioritized in future development policies of the Chinese government. Nevertheless, China’s agricultural sector has proven to be quite resilient; its total grain production has increased for the past 11 consecutive years. While many factors have contributed to this, agricultural-production outsourcing (a relatively new institution) is believed to have been significant in recent years; however, the associated determinants and consequences of agricultural-production outsourcing remain poorly understood. This is one of the very few papers exploring this important and emerging issue. Our econometrics results show that farm scale, labor endowment, government subsidies, and the ownership of agricultural machinery are key determinants in the decisions of rice farmers to outsource production tasks.

This has yielded a number of important policy implications. 1) The benefits associated with large farm scale are supported by the finding that the relatively small scale (of farms) can significantly limit the interest of rice farmers in outsourcing agricultural production; these may be attained via the land rental market. For this reason, local governments should (i) remove any restrictions on land rental transfers, (ii) guarantee security if the land is rented out to others, and (iii) reduce fragmentation (if an opportunity is presented to reallocate land during a new titling process). 2) Subsidies appear to be an effective mechanism for promoting agricultural outsourcing; however, they should target tasks that are less widely adopted and thus more responsive to subsidies. For example, the outsourcing of harvesting is already widely adopted; thus, government subsidies are likely to have a limited effect on promoting the outsourcing of this task any further. Relatedly, it may be advisable for local governments to emphasize the supply of outsourcing services in regions where, for example, ownership of agricultural machines is relatively limited and labor migration is relatively active (since ownership of agricultural machinery and labor endowment constraints are important determinants of the adoption of outsourcing services).

Our study does have some limitations. 1) The main limitation is the lack of panel data; due to this, our analysis focuses on associations between determinants and adoption behaviors (of outsourcing services). Panel data are also needed for future studies on the impact of agricultural outsourcing services on productivity and household welfare. 2) Also, we would have preferred more reliable pricing data for outsourcing services (e.g., task-specific prices). 3) In future research, we would also like to have more detailed data on migrations at the member level (vs. exclusively on heads of households). Future research on the effects of agricultural outsourcing on technical efficiencies would help guide the debate on agricultural outsourcing services on production and food security.
